# Quantification of left ventricular trabeculae using cardiovascular magnetic resonance for the diagnosis of left ventricular non-compaction: evaluation of trabecular volume and refined semi-quantitative criteria

**DOI:** 10.1186/s12968-016-0245-2

**Published:** 2016-05-04

**Authors:** Yeonu Choi, Sung Mok Kim, Sang-Chol Lee, Sung-A Chang, Shin Yi Jang, Yeon Hyeon Choe

**Affiliations:** Department of Radiology, Samsung Medical Center, Sungkyunkwan University School of Medicine, 81 Irwon-ro, Gangnam-gu, Seoul, 06351 Korea; HVSI Imaging Center, Heart Vascular Stroke Institute, Samsung Medical Center, Sungkyunkwan University School of Medicine, Seoul, Korea; Division of Cardiology, Department of Medicine, Samsung Medical Center, Sungkyunkwan University School of Medicine, Seoul, Korea

**Keywords:** Left ventricle, Non-compaction, Cardiovascular magnetic resonance, Trabeculated left ventricular volume

## Abstract

**Background:**

Left ventricular non-compaction (LVNC) is an unclassified cardiomyopathy and there is no consensus on the diagnosis of LVNC. The aims of this study were to establish quantitative methods to diagnose LVNC using cardiovascular magnetic resonance (CMR) and to suggest refined semi-quantitative methods to diagnose LVNC.

**Methods:**

This retrospective study included 145 subjects with mild to severe trabeculation of the left ventricle myocardium [24 patients with isolated LVNC, 33 patients with non-isolated LVNC, 30 patients with dilated cardiomyopathy (DCM) with non-compaction (DCMNC), 27 patients with DCM, and 31 healthy control subjects with mild trabeculation]. The left ventricular (LV) ejection fraction, global LV myocardial volume, trabeculated LV myocardial volume, and number of segments with late gadolinium enhancement were measured. In addition, the most prominent non-compacted (NC), compacted (C), normal mid-septum, normal mid-lateral wall, and apical trabeculation thicknesses on the end-diastolic frames of the long-axis slices were measured.

**Results:**

In the patients with isolated LVNC, the percentage of trabeculated LV volume (TV%, ​42.6 ± 13.8 %) ​relative to total LV myocardial volume was 1.4 times higher than in those with DCM (30.3 ± 14.3 %, *p* < 0.001), and 1.7 times higher than in the controls (24.8 ± 7.1 %, *p* < 0.001). However, there was no significant difference in TV% between the isolated LVNC and DCMNC groups (47.1 ± 17.3 % in the DCMNC group; *p* = 0.210). The receiver operating characteristic curve analysis using Jenni’s method for CMR classification as the standard diagnostic criteria revealed that a value of TV% above 34.6 % was predictive of NC with a specificity of 89.7 % (CI: 74.2 - 98.0 %) and a sensitivity of 66.1 % (CI: 52.6 - 77.9 %). A value of NC/septum over 1.27 was considered predictive for NC with a specificity of 82.8 % (CI: 64.2 - 94.2 %) and a sensitivity of 57.6 % (CI: 44.1 - 70.4 %). In addition, a value of apex/C above 3.15 was considered predictive of NC with a specificity of 93.1 % (CI: 77.2 - 99.2 %) and a sensitivity of 69.5 % (CI: 56.1 - 80.8 %).

**Conclusions:**

A trabeculated LV myocardial volume above 35 % of the total LV myocardial volume is diagnostic for LVNC with high specificity. Also, the apex/C and NC/septum ratios could be useful as supplementary diagnostic criteria.

## Background

Left ventricular non-compaction (LVNC) is an unclassified cardiomyopathy characterized by an extremely thick endocardial layer with prominent trabeculation and a thin epicardial layer [1]. LVNC has been linked to several genetic mutations and, in adult forms of LVNC, it is predominantly an autosomal-dominant inheritance pattern [2, 3]. Although LVNC can occur in the absence of other coexisting cardiac abnormalities [4], it can also be associated with various forms of congenital heart diseases, particularly stenotic lesions of the left ventricular outflow tract, Ebstein’s anomaly, and tetralogy of Fallot [5], and in addition, there are some relationships between LVNC and neuromuscular disorders [6]. It is widely known that the major clinical manifestations of LVNC are heart failure, thrombo-embolism, and arrhythmia [7, 8].

There have been many attempts to establish standard diagnostic criteria in LVNC using various imaging modalities. At present, Jenni et al.’s echocardiographic criteria of a ratio over 2.0 between the thickness of the non-compacted and compacted myocardial layers in systole are widely recognized as the standard diagnostic criteria for LVNC [4]. Echocardiography is useful for the diagnosis of LVNC; however, there are concerns about overestimated diagnosis of LVNC due to the high sensitivity of the echocardiographic criteria [9, 10]. Recently, cardiovascular magnetic resonance (CMR) is becoming more widely used in the assessment of LVNC [11]. CMR allows more accurate and reliable evaluation of the extent of non-compacted myocardium than does two-dimensional echocardiography and provides supplementary morphological information, particularly in the left ventricle (LV) apex and lateral wall [12]. Petersen et al. suggested a semi-quantitative method to diagnose LVNC using CMR [13]. They assessed each of three diastolic long-axis cine MR images, and a maximum ratio of non-compacted to compacted myocardial thicknesses (NC/C ratio) greater than 2.3 is considered diagnostic for LVNC. However, LVNC with apical involvement could not be assessed with their criteria because of the thin apical compact myocardium. Jacquier et al. proposed a quantitative method to diagnose LVNC by measuring trabeculated LV mass [14]. The trabeculated LV mass was calculated by subtracting the compacted LV mass from the total LV mass. Based on this, a percentage of trabeculated LV mass over 20 % was considered as the diagnostic cut-off.

Even when using high-contrast imaging modalities such as CMR, however, there are still concerns about the overdiagnosis of LVNC [15, 16]. In addition, there has been no consensus in the diagnosis of LVNC. Therefore, the purposes of this study were to determine quantitative diagnostic criteria for LVNC using CMR and to suggest refined semi-quantitative methods to diagnose LVNC, especially in cases with apical involvement.

## Methods

This study was approved by the Samsung Medical Center Institutional Review Board; informed consent was waived for this retrospective study.

### Study population

We queried the clinical and CMR databases at our institute for patients diagnosed with cardiomyopathy between August 2009 and December 2013. Of a total of 11,997 patients diagnosed with cardiomyopathy, we extracted consecutive patients who had CMR reports that included descriptions of non-compaction. We also included subjects with mildly or moderately increased LV trabeculation (hypertrabeculation) measuring more than 5 mm in thickness and NC/C ratios of 1.0–2.3 anywhere in the myocardial segments on the CMR images.

We enrolled the LVNC group according to two inclusion criteria: CMR images with a distinct two-layered appearance of trabeculated and compacted myocardium and the fulfillment of Petersen et al.’s CMR criteria for the diagnosis of LVNC. As dilated cardiomyopathy (DCM) is a potential differential diagnosis for LVNC, we retrospectively enrolled two other groups: DCM with LVNC and DCM with hypertrabeculation. We classified patients with DCM into two groups according to Petersen et al.’s CMR criteria. The diagnosis of DCM was made on the basis of impaired global LV function with an ejection fraction of less than 40 % on CMR, LV chamber dilatation, and the exclusion of other causes of LV dysfunction.

The remaining subjects were a healthy control group without a history of cardiovascular symptoms, valvular heart disease, coronary artery disease, or cardiomyopathy. We randomly extracted sex- and age-matched subjects for the control group. Finally, the study population comprised of a total of 145 patients divided into the following five subgroups: patients for whom a diagnosis of LVNC was established based on Petersen et al.’s CMR criteria (group 1 for isolated LVNC [INC], *n* = 24; group 2 for accompanied with other diseases [non-isolated type, NINC], *n* = 33; group 3 for patients with DCM with LVNC [DCMNC], *n* = 30; group 4 for patients with DCM and hypertrabeculation, *n* = 27; and group 5 for the healthy control group with hypertrabeculation, *n* = 31) (Table 1).

**Table 1:** Patient characteristics in five groups

Variables	Isolated LVNC (*n* = 24)	Non-isolated LVNC (*n* = 33)	DCMNC (*n* = 30)	DCM (*n* = 27)	Control (*n* = 31)
Age, years	51.2 ± 12.8	55.1 ± 16.5 (*P* = 0.179)	53.0 ± 14.1 (*P* = 0.688)	59.2 ± 15.8 (*P* = 0.026)	55.4 ± 7.8 (*P* = 0.137)
Sex, male (%)	50.0	57.6	60.0	70.4	74.2
Height, cm	164.7 ± 7.5	167.0 ± 9.0 (*P* = 0.303)	162.3 ± 8.2 (*P* = 0.280)	164.4 ± 10.5 (*P* = 0.962)	166.2 ± 8.7 (*P* = 0.518)
Weight, kg	62.9 ± 12.3	65.2 ± 10.7 (*P* = 0.593)	63.4 ± 13.7 (*P* = 0.896)	63.3 ± 14.1 (*P* = 0.992)	68.0 ± 12.2 (*P* = 0.166)
BSA, m^2^	1.68 ± 0.19	1.73 ± 0.17 (*P* = 0.460)	1.67 ± 0.18 (*P* = 0.501)	1.69 ± 0.23 (*P* = 0.879)	1.76 ± 0.19 (*P* = 0.205)
SBP, mmHg	117.9 ± 11.6	118.0 ± 17.2 (*P* = 0.935)	114.0 ± 18.7 (*P* = 0.061)	113.0 ± 15.1 (*P* = 0.274)	127.9 ± 13.0*
DBP, mmHg	66.7 ± 8.4	67.7 ± 10.8 (*P* = 0.446)	68.8 ± 11.3 (*P* = 0.500)	67.6±12.0 (*P* = 0.992)	75.3 ± 8.5*
TC, mg/dL	195.6 ± 46.6	168.8 ± 30.6 (*P* = 0.028)	170.7 ± 41.2 (*P* = 0.023)	165.1±35.2 (*P* = 0.020)	197.1 ± 33.0 (*P* = 0.812)
TG, mg/dL	148.5 ± 147.0	117.1 ± 59.2 (*P* = 0.992)	125.5 ± 99.1 (*P* = 0.726)	118.0±82.2 (*P* = 0.725)	125.6 ± 72.1 (*P* = 0.926)
LDL-C, mg/dL	119.6 ± 36.0	104.9 ± 33.3 (*P* = 0.114)	105.7 ± 28.9 (*P* = 0.105)	94.7 ± 27.5 (*P* = 0.018)	127.0 ± 28.4 (*P* = 0.441)
HDL-C, mg/dL	58.7 ± 19.6	51.0 ± 12.2 (*P* = 0.166)	46.5 ± 15.2 (*P* = 0.038)	52.3 ± 23.9 (*P* = 0.080)	53.0 ± 14.1 (*P* = 0.244)
FBS, mg/dL	99.3 ± 11.7	104.9 ± 20.1 (*P* = 0.321)	122.6 ± 52.6 (*P* = 0.052)	115.0 ± 45.3 (*P* = 0.253)	101.1 ± 14.6 (*P* = 0.634)
Hypertension (%)	12.5	18.2 (*P* = 0.720)	26.7 (*P* = 0.310)	25.9 (*P* = 0.300)	22.6 (*P* = 0.486)
Diabetes (%)	8.3	15.2 (*P* = 0.687)	20.0 (*P* = 0.277)	33.3**	6.5 (*P* = 1.000)
Dyslipidemia (%)	41.7	33.3 (*P* = 0.585)	16.7 (*P* = 0.066)	18.5 (*P* = 0.123)	45.2 (*P* = 1.000)
CHF (%)	0	18.2**	46.7**	59.3**	0.0
CAD (%)	0	18.2 **	3.3 (*P* = 1.000)	7.4 (*P* = 0.492)	3.2 (*P* = 1.000)
CVA/TIA (%)	0	0	3.3 (*P* = 1.000)	11.1 (*P* = 0.238)	0.0
Arrhythmia (%)	0	30.3**	20.0**	18.5 (*P* = 0.052)	0.0

Note- Data are mean ± standard deviation (SD)

*BSA* body surface area, *CAD* coronary artery disease, *CHF* congestive heart failure, *CVA* cerebrovascular accident, *DBP* diastolic blood pressure, *FBS* fasting blood sugar, *HDL* high density lipoprotein-cholesterol, *LDL* low density lipoprotein-cholesterol, *SBP* systolic blood pressure, *TC* total cholesterol, *TG* triglyceride, *TIA* transient ischemic attack. P-value was calculated in comparison with values obtained from isolated LVNC patients and *p < 0.0125, **p < 0.05

The distribution of the percentages of trabeculated LV volumes and the various ratios in the long-axis cine images were studied in the five different groups. To ensure the efficient measurement of the LV trabeculated areas, we excluded two subjects with low CMR image quality due to arrhythmic or respiratory artifacts. All clinical and demographic data were obtained from the electronic medical records.

### Characteristics of patients

We reviewed the electronic medical records of the patients for diabetes, hypertension, dyslipidemia, congestive heart failure (CHF), coronary artery disease (CAD), and any arrhythmias. Diabetes was defined as a patient who had been diagnosed with diabetes or was taking glucose-lowering medication. Hypertension was defined as a diagnosis of hypertension or patients with systolic blood pressure ≥ 140 mmHg or diastolic blood pressure ≥ 90 mmHg. Dyslipidemia was defined by laboratory findings (total cholesterol > 200 mg/dL or low-density lipoprotein cholesterol > 130 mg/dL). To assess arrhythmia, we reviewed the patients’ electrocardiographic findings, and any arrhythmia from atrial fibrillation to ventricular tachycardia was included.

### Acquisition of CMR data

**Fig. 1:**
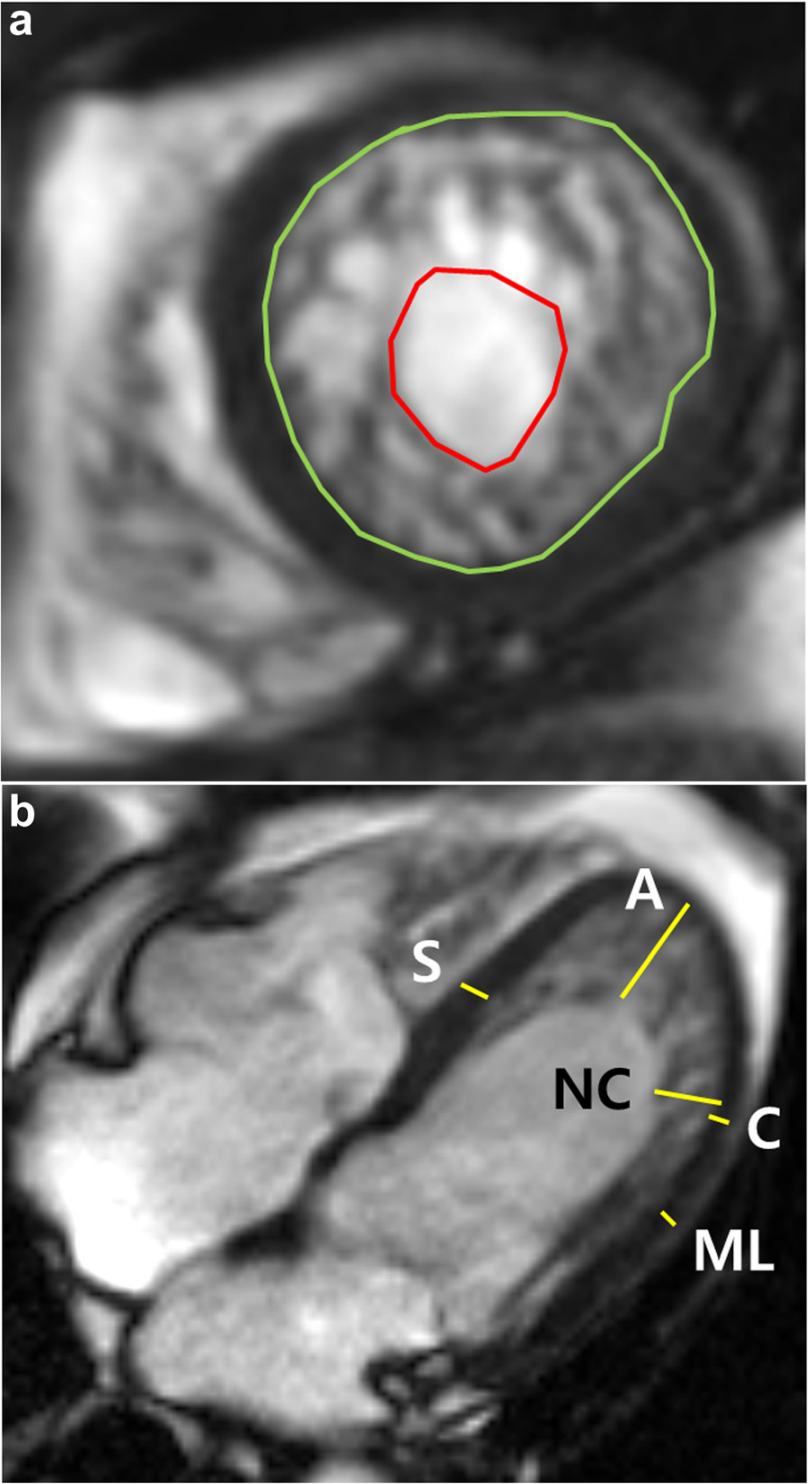
Measurement of left ventricle non-compaction (LVNC) with CMR. Illustration of the described method for measuring the trabeculated LV area in a patient with isolated LVNC. The trabeculated LV area was measured manually using a picture archiving and communication system on an end-diastolic frame of each short-axis slice (**a**). An endocardial border and non-compacted layer border were drawn to include the trabeculated area, and the papillary muscles were excluded from the measurement. We measured the most prominent non-compacted to compacted ratio in three long-axis views and also measured the mid-septal and mid-lateral walls and the apical trabeculation thickness in diastole; all measurements were performed perpendicular to the epicardium (**b**). A, apical trabeculation; C, compacted myocardium for Petersen’s criteria; ML, mid-lateral wall; NC, non-compacted area for Petersen’s criteria; S, mid-septal wall

All patients underwent cardiac MRI using a 1.5 T scanner (Magnetom Avanto, Syngo MR version B17; Siemens Medical Solutions, Erlangen, Germany) with a 32-channel phased-array receiver coil during repeated breath-holds. After localization, cine images of LV were acquired using a steady-state free-precession sequence in four-, two-, and three-chamber views and in short-axis views to obtain 20–30 contiguous short-axis slices to include the entire LV with a slice thickness of 6 mm and gaps of 4 mm (Fig. 1). In cases with arrhythmia or breathing difficulty, fast cine MRI with a temporal parallel acquisition technique (acceleration factor: 3) was used for cine MRI.

Standard late gadolinium-enhanced imaging was performed using a phase-sensitive inversion-recovery technique 15 min after an injection of 0.2 mmol/kg gadobutrol (Gadovist; Bayer Healthcare, Berlin, Germany) using contiguous short-axis image acquisition of 10 to 12 slices at 6-mm thickness with 4-mm interslice gaps. Inversion delay times were typically 280 to 360 msec.

### CMR analysis

#### Non-compaction distribution analysis

The distribution of LVNC was assessed by the qualitative analysis of all 17 American Heart Association segments for the presence of a distinct two-layered appearance. A segment was regarded as having hypertrabeculation if the trabeculated to compacted ratio (NC/C ratio) in diastole was higher than 1.0. In addition, an NC/C ratio over 2.3 is considered as non-compaction. We excluded segments where the trabeculated area was less than 50 % of the total thickness.

We also classified the distribution of trabeculation into three patterns: the global, apical, and non-apical types. The global type was defined as having over ten two-layered segments in total. The apical type was defined as involving more than three segments in the apical level (segments 13 through 17). The non-apical type was any trabeculation that did not meet the definitions of the global or apical types.

#### Semi-quantitative measurement

During the measurement of the most prominent trabeculation to compacted ratio in three long-axis views as in Petersen’s CMR criteria [13], we also measured mid-septum and mid-lateral wall thicknesses in diastole. If apical trabeculation was present, the trabeculated thickness was measured perpendicular to the apex in diastole. We calculated the ratios of the thickness of apical trabeculation to that of compacted myocardium in the apical lateral segments (apex/C ratio), the apical trabeculation to the septal wall thickness (apex/septum ratio), the apical trabeculation to the mid-lateral wall thickness (apex/mid-lateral ratio), and the trabeculated myocardium to the mid-septal wall thickness (NC/septum ratio) (Fig. 1). We calculated ratios from each of the three long-axis views, and only the maximal ratio was then used for analysis. The end-systolic ratio of non-compacted to compacted myocardial thickness was also measured on short-axis cine images in systole.

#### Trabeculated volume measurement

Using short-axis cine images, we determined end-diastolic frames when the cavity sizes were largest and end-systolic frames when the cavity sizes were smallest. Trabeculated volume measurements were performed on the end-diastolic frames of each short-axis slice in the LV stack. After we identified a distinct two-layered structure, we manually measured the trabeculated area with a picture archiving and communication system (Centricity™ PACS, General Electric Healthcare Integrated IT Solutions, Barrington, IL, USA). An endocardial border and non-compacted layer border were drawn to include the trabeculated area, and the papillary muscles were specifically excluded from the measurement (Fig. 1). When the papillary muscles were indistinguishable from trabeculation, they were treated as trabeculation. The percentage of trabeculated LV volume (TV%) relative to total LV myocardial volume was calculated by the following equation: ([trabeculated LV volume] ∕ [total LV myocardial volume]) × 100. The trabecular volume index was calculated by dividing the trabecular volume by the body surface area (BSA).

The LV ejection fraction (LVEF), LV end-diastolic volume (LVEDV), LV end-systolic volume (LVESV) and LV myocardial mass were measured using an Argus workstation (Siemens). LVEDVi was calculated as LVEDV/BSA and LVESVi was calculated as LVESV/BSA.

Wall thickening of compact myocardium in the non-compaction areas was measured from the short-axis cine images of the apical level using the following equation: compact myocardial wall thickening = ([end-systolic compact wall thickness] − [end-diastolic compact wall thickness])/[end-diastolic compact wall thickness].

We also assessed the number of myocardial segments with late gadolinium enhancement (LGE). LGE was defined as a signal intensity higher than three standard deviations above the mean intensity of the region of interest in the remote normal myocardium using semi-quantitative software (QMass ES, Medis Medical Imaging Systems, Leiden, The Netherlands).

### Statistical analysis

All continuous data are presented as means ± standard deviations (SDs), and categorical data are presented as percentages. The statistical analysis was performed using SPSS for Windows 18.0 (IBM, Chicago, IL, USA).

Continuous data in the patient group characteristics were compared by the Kruskal–Wallis non-parametric tests; when results from these were significant, we compared them by the Mann–Whitney U non-parametric tests for post hoc analysis. In these cases, the Bonferroni correction was applied to ensure an overall type I error rate of 5 % and an adjusted *P* < 0.05/4 = 0.0125 was considered significant. In addition, the categorical data in the patient group characteristics were compared by chi-square tests.

The interobserver reproducibility was assessed using intraclass correlation and Bland–Altman analysis [17] by calculating the bias (mean difference) and the 95 % limits of agreement (1.96 times the SD around the mean difference). To assess interobserver and intraobserver consistency, a subset of cases (*n *= 20) was randomly selected and measured by a second rater blinded to all information.

We used receiver operating characteristics (ROC) curves to determine the optimal cut-off values of the trabeculated volume, apex/C, apex/septum, apex/mid-lateral, and NC/septum ratios. A comparison of the areas under the ROC curves was performed using the method described by Hanley et al. [18]. Based on the ROC curve analysis, we determined the optimal diagnostic thresholds of each value to distinguish the highly trabeculated myocardium (LVNC groups) from patients with a normal amount of trabeculation (healthy control group). As the frequency of LGE-positive segments was high in the DCMNC and DCM groups, we analyzed the correlation between the number of LGE segments and trabeculated LV volume in the DCMNC and DCM groups.

## Results

### Characteristics of patients

The study population was comprised of 145 patients and was homogeneous in its race and ethnicity, Asian (Korean). All four groups were matched in terms of age and sex, and all groups were also comparable in terms of height, weight, and body surface area (Table 1).

The proportions of hypertension, diabetes, and dyslipidemia did not differ significantly among the five groups (Table 1). However, there were statistically different proportions of CAD, CHF, arrhythmias, cerebrovascular accidents (CVA), and transient ischemic attacks (TIA) among the five groups. Compared with the other three groups, the DCMNC and DCM patients had greater incidences of diabetes (20–33 % versus 7–15 %), CHF (47–59 % versus 0–18 %), and CVA/TIA (3–11 % versus 0 %). In contrast, dyslipidemia was more common in the LVNC and control groups than in the DCMNC and DCM groups (33–45 % versus 17–19 %). Arrhythmia was more common in the NINC, DCMNC and DCM groups as compared with the INC and control groups (18–30 % versus 0 %). However, there were no statistically significant differences in the incidence of arrhythmia among the three groups.

In the INC group, only one patient among 24 was diagnosed as having myotonic dystrophy. In the patients with LVNC and accompanying heart diseases, there were CADs including myocardial infarction (old, 9; acute, 1), valvular heart diseases (*n* = 8; aortic regurgitation = 5, mitral regurgitation = 3), arrhythmia (*n* = 10), congenital heart diseases (*n* = 3), unclassified cardiomyopathy (*n* = 3), hypertrophic cardiomyopathy (*n* = 2), and Marfan syndrome (*n* = 1). We had four LVNC patients with ventricular arrhythmia (ventricular fibrillation = 1, ventricular tachycardia = 3) not associated other structural heart diseases.

CAD, CHF, and arrhythmia were significantly more frequent in the NINC group than in the INC group (CAD, 18.2 % versus 0 %; CHF, 18.2 % versus 0 %; arrhythmia, 30.3 % versus 0 %; Table 1).

There was no significant difference in N-terminal pro-brain natriuretic peptide (NT-proBNP) between INC group (40.5 ± 38.8 pg/mL) and control group (34.8 ± 29.8 pg/mL) by Student *t*-test (*p* = 0.535).

### Distribution of non-compacted area

**Fig. 2:**
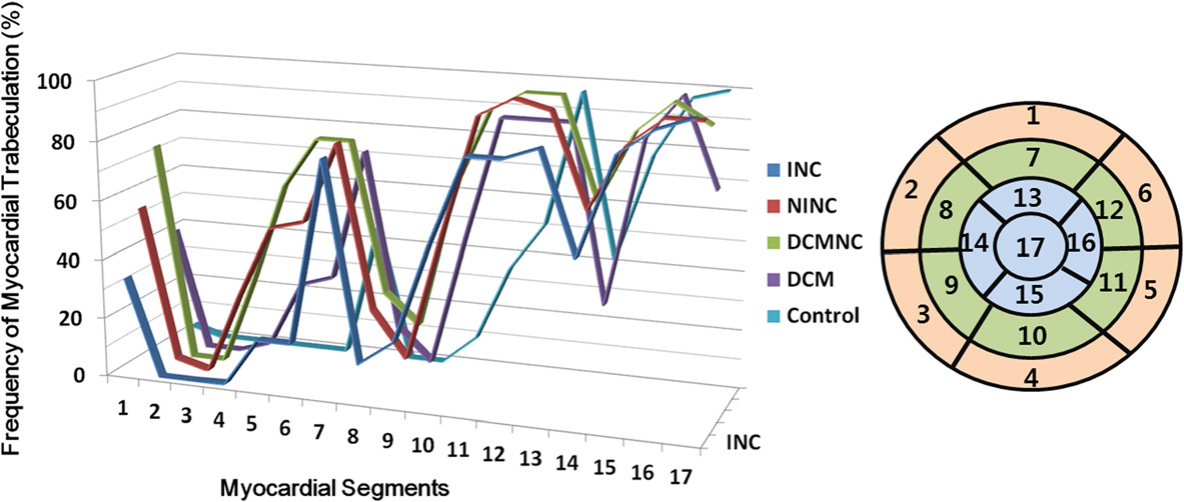
Distribution of left ventricular trabeculation (NC/C > 1.0 and trabeculation thickness > 5 mm) in all the patient groups according to the American Heart Association 17-segment model (bull’s eye diagram). Control, normal subjects with hypertrabeculation; DCM, dilated cardiomyopathy with hypertrabeculation; DCMNC, dilated cardiomyopathy with non-compaction; INC, isolated non-compaction; NINC, non-isolated non-compaction

The patterns of distribution of the trabeculation areas with trabeculation thicknesses of more than 5 mm in the American Heart Association’s 17-segment model were almost similar among the groups. There were more basal segments in the NINC group (54.5 %) than in the INC group (33.3 %) (Fig. 2). In the control group, the trabeculation involved less basal segments than in the other groups. Also, the DCM group showed less apical segment (70.4 %) involvement than other groups (93.3 % - 100 %) did.

**Fig. 3:**
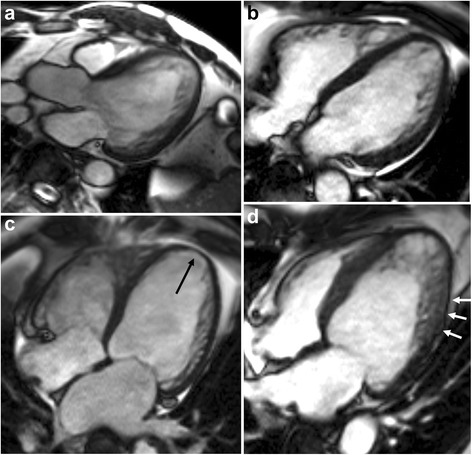
Types of LV non-compaction or hypertrabeculation on long-axis cine MR images. **a** Global type in a patient with isolated LVNC, **b** apical type in a patient with non-isolated LVNC, c) non-apical type in a patient with DCM, d) global type with severe wall thinning (arrows) in the compacted myocardium. Note absence of trabeculation in the apex (arrow) in the non-apical type (**c**)

In our classification of the distribution of trabeculation (NC/C > 1.0 and trabeculation thickness > 5 mm) in 57 LVNC patients, 25 were the global type (43.9 %), 30 were the apical type (52.6 %), and two were the non-apical type (3.5 %). Of the 30 DCMNC cases, 22 were the global type (73.3 %) and eight were the apical type (26.7 %). Of the 27 DCM cases, five were the global type (18.5 %), 19 were the apical type (70.4 %), and three were non-apical type (11.1 %). Finally, of the 31 control cases, one was the global type (3.2 %) and 30 were the apical type (96.8 %) (Fig. 3). Interestingly, trabeculation was absent in the LV apex (segment 17) in 9 (6.2 %) of 145 subjects (Fig. 3). Six (22.2 %) DCM patients, two (6.7 %) DCMNC patients, and one (3.0 %) NINC patient showed no apical trabeculation.

**Fig. 4:**
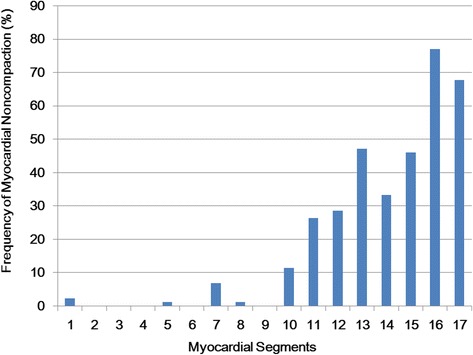
Segmental distribution of LVNC (NC/C > 2.3) in 87 patients with LVNC. LVNC is most frequent in the apical segments and also distributed in the inferior and lateral segments of the middle level of LV

In 87 patients with LVNC (groups of INC, NINC, DCMNC), severe trabeculation meeting Peterson’s criteria of LVNC (NC/C > 2.3) was observed most frequently in the segment 16 (77 %) followed by the segment 17 (67.8 %), segment 13 (47.1 %), segment 15 (46 %), segment 12 (28.7 %), segment 11 (26.4 %), segment 10 (11.4 %), segment 7 (6.9 %), segment 1 (2.3 %), segment 8 (1.1 %), and segment 5 (1.1 %) (Fig. 4).

### Semi-quantitative non-compaction criteria, LV volumes, and LGE according to groups

In patients with LVNC, the NC/C ratios measured by Petersen’s method [13] were significantly higher than those in the DCM and control groups (Table 2). Also, the same pattern was seen in the NC/septum, apex/C, apex/septum, and apex/mid-lateral ratios. In addition, all five of the ratios that we measured were statistically similar between LVNC and DCMNC groups.

**Table 2:** CMR measurements of left ventricle function, trabecular volume, and semi-quantitative diagnostic criteria

Variables	Isolated LVNC (*n* = 24)	Non-isolated LVNC (*n* = 33)	DCMNC (*n* = 30)	DCM (*n* = 27)	Control (*n* = 31)
LVEF, %	64.7 ± 6.4	47.6 ± 17.0*	24.8 ± 9.2*	27.9 ± 10.1*	66.5 ± 5.9 (*P* = 0.430)
LVEDV, mL	131.1 ± 28.5	186.8 ± 60.9*	293.0 ± 120.2*	277.0 ± 92.7*	134.4 ± 22.9 (*P* = 0.722)
LVEDVi, mL/m^2^	77.6 ± 12.9	107.8 ± 33.6*	173.0 ± 56.4*	165.9 ± 57.8*	76.5 ± 8.8 (*P* = 0.754)
LVESV, mL	47.1 ± 17.0	101.0 ± 54.0*	225.6 ± 110.7*	203.9 ± 83.1*	45.5 ± 12.4 (*P* = 1.000)
LVESVi, mL/m^2^	27.7 ± 8.3	58.6 ± 31.2*	132.8 ± 53.9*	122.0 ± 51.0*	25.7 ± 5.9 (*P* = 0.635)
SV, mL	84.0 ± 16.7	85.8 ± 32.7 (p = 0.518)	67.4 ± 30.7*	73.0 ± 29.2 (*P* = 0.045)	89.0 ± 14.5 (*P* = 0.235)
NC/C ratio^a^	3.08 ± 0.80	2.90 ± 0.53 (p = 0.593)	2.92 ± 0.77 (*P* = 0.342)	1.71 ± 0.34*	1.40 ± 0.39*
NC/septum ratio	1.45 ± 0.28	1.37 ± 0.34 (p = 0.304)	1.52 ± 0.44 (*P* = 0.566)	1.07 ± 0.29*	0.93 ± 0.22*
Apex/C ratio	4.84 ± 1.89	3.57 ± 1.89 (p = 0.056)	3.26 ± 1.80*	1.25 ± 1.15*	1.96 ± 1.01*
Apex/septum ratio	2.28 ± 0.84	1.69 ± 0.96 (*P* = 0.023)	1.71 ± 0.95 (*P* = 0.065)	0.83 ± 0.89*	1.31 ± 0.73*
Apex/mid-lat ratio	4.53 ± 2.51	3.00 ± 1.70 (*P* = 0.048)	2.99 ± 1.69 (*P* = 0.049)	1.24 ± 1.18*	1.89 ± 0.99*
Trab. Vol., mL	32.1 ± 13.8	45.1 ± 15.3*	61.6 ± 25.5*	41.9 ± 18.4*	23.9 ± 6.9*
Trab. Vol., %LV	42.6 ± 14.8	44.2 ± 15.4 (p = 0.891)	47.1 ± 17.3 (*P* = 0.210)	30.3 ± 14.3*	24.8 ± 7.1*

*C* compacted, *apex* apical trabeculation thickness, *LVNC* left ventricular non-compaction, *DCM* dilated cardiomyopathy, *DCMNC* DCM with left ventricular non-compaction, *LVEF* left ventricular ejection fraction, LVEDV left ventricular end-diastolic volume, *LVESV* left ventricular end-systolic volume, *mid-lat* mid-lateral wall thickness, *NC* non-compacted, *SV* stroke volume, *septum* septal wall thickness, *Trab*. trabeculated, *vol.* volume. Values are mean ± SD. *P*-value was calculated in comparison with values obtained from LVNC patients. *P < 0.013

^a^Results from analysis by the Petersen’s method

In INC group, LVEDVi (77.6 ± 12.9 mL/m^2^) was 2.2 times lower than in DCMNC group (173.0 ± 56.4 mL/m^2^), and similar to the control group (76.5 ± 8.8 mL/m^2^). In INC group, LVESVi (27.7 ± 8.3 mL/m^2^) was 4.8 times lower than in DCMNC group (132.8 ± 53.9 mL/m^2^), and similar to the control group (25.7 ± 5.9 mL/m^2^) (Table 2).

**Fig. 5:**
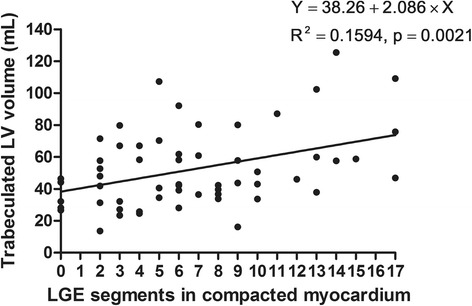
Scattergrams of trabeculated LV volumes against late gadolinium enhancement (LGE) segments in the compacted myocardia of patients with dilated cardiomyopathy (DCM) with hypertrabeculation and dilated cardiomyopathy with non-compaction (DCMNC). Simple linear regression lines were applied to demonstrate relationships. Trabeculated LV volume was positively correlated with the number of LGE-positive segments (Spearman ρ, 0.350; *p* = 0.008)

The proportion of patients with LGE-positive segments was 4.2 % (1/24) in the INC group. In the NINC group, however, 45.5 % (15/33) of the patients had LGE-positive segments. The mean number of segments with LGE was 0.04 ± 0.20 in INC group. However, there were statistically higher mean numbers of LGE-positive segments in the NINC (2.7 ± 3.9, *p* < 0.001), DCMNC (7.1 ± 5.1, *p* < 0.001), and DCM (6.3 ± 4.1, *p* < 0.001) groups. The trabeculated LV volume was positively correlated with the number of LGE-positive segments (Spearman ρ: 0.350; *p* = 0.008) (Fig. 5). In a subgroup analysis, the DCMNC group showed positive correlation between the number of LGE segments and trabeculated LV volume (Spearman ρ: 0.396; *p* = 0.03). In the DCM group, however, there was no correlation between the number of LGE segments and trabeculated LV volume.

### Correlation between trabeculated LV volume and LV function measurements

**Fig. 6:**
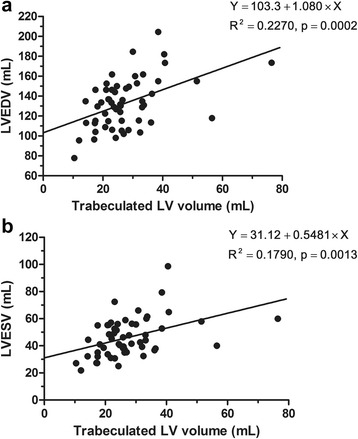
Scattergrams of trabeculated LV volume versus left ventricular end-diastolic volume (LVEDV) (**a**) and left ventricular end-systolic volume (LVESV) (**b**) in subjects from the isolated left ventricle non-compaction (INC) group and the healthy control group. Simple linear regression lines were applied to demonstrate relationships. Trabeculated volume correlated positively with LVEDV (Spearman ρ, 0.591; *p* = 0.006) and LVESV (Spearman ρ, 0.618; *p* = 0.004)

In analyses of the INC group and the control group, which had no other cardiac abnormalities, trabeculated volume correlated positively with LVEDV (Spearman ρ: 0.591; *p* = 0.006) and LVESV (Spearman ρ: 0.618; *p* = 0.004) (Fig. 6). However, no correlation emerged between trabeculated LV volume and LVEF in INC and control groups.

**Fig. 7:**
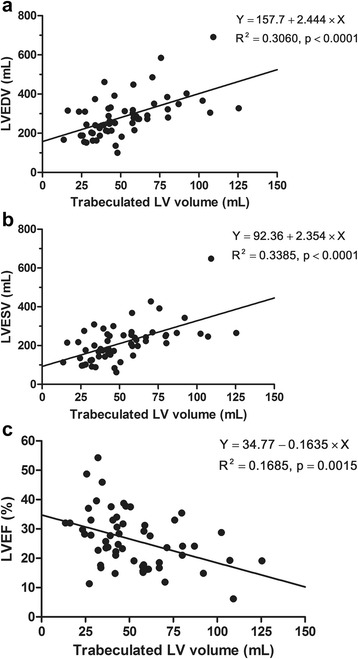
Scattergrams of trabeculated LV volume versus left ventricular end-diastolic volume (LVEDV) (**a**), left ventricular end-systolic volume (LVESV) (**b**), and left ventricular ejection fraction (LVEF) (**c**) in patients from the dilated cardiomyopathy with hypertrabeculation (DCM) and the dilated cardiomyopathy with non-compaction (DCMNC) groups. Simple linear regression lines were applied to demonstrate relationships. Trabeculated LV volume correlated positively with LVEDV (Spearman ρ, 0.556; *p* < 0.001) and LVESV (Spearman ρ, 0.555; *p* < 0.001) and negatively with LVEF (Spearman ρ, −0.385; *p* = 0.003)

In the DCMNC and DCM groups, trabeculated LV volume correlated positively with LVEDV (Spearman ρ: 0.556; *p* < 0.001) and LVESV (Spearman ρ: 0.555; *p* < 0.001) and negatively with LVEF (Spearman ρ: −0.385; *p* = 0.003) (Fig. 7). In results that were re-analyzed by separating the groups, the trabeculated LV volume was positively correlated with LVEDV (Spearman ρ: 0.755; *p* < 0.001) and LVESV (Spearman ρ: 0.753; *p* < 0.001) and negatively correlated with LVEF (Spearman ρ: −0.423; *p* = 0.02) in the DCMNC group. In the DCM group, however, there were no significant correlations except with LVEDV (Spearman ρ: 0.439; *p* = 0.022).

The percentage of trabeculated LV myocardial volume was not significantly correlated with LVEF, LVEDV, or LVESV in any of the groups. In the INC group, LVEF under 55 % was found in only 8.3 % (2/24) of subjects.

The thickness of compacted myocardium in the area of LVNC or hypertrabeculation was less than 2 mm in 91.7 % (22/24) of INC, 66.7 % (22/33) of NINC, 86.7 % (26/30) of DCMNC, 70.4 % (19/27) of DCM, 9.7 % (3/31) of control group (Fig. 3). There was no significant difference in mean compacted wall thickening among INC (0.77 ± 0.66), NINC (0.77 ± 0.51) and DCMNC (0.58 ± 0.58) (*p* = 0.3478) groups by one-way analysis of variance (ANOVA).

### Distribution of the percentage of trabeculated LV volume and reproducibility

**Fig. 8:**
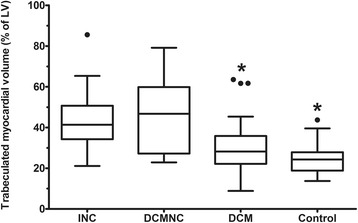
Distribution of the trabeculated myocardial volume over left ventricular myocardial volume (%) for each group. The boxes extend from the 25th percentile to the 75th percentile, and the whiskers extend to 1.5 times the interquartile distance. Control, normal subjects with hypertrabeculation; DCM, dilated cardiomyopathy with hypertrabeculation; DCMNC, dilated cardiomyopathy with non-compaction; INC, isolated LV non-compaction. P-values were calculated in comparison with the values obtained in the LVNC patients (*p < 0.013)

In the patients with INC, TV% (42.6 ± 13.8 %) was 1.4 times higher than that in DCM (30.3 ± 14.3 %, *p* < 0.001), and 1.7 times higher than that in the controls (24.8 ± 7.1 %, *p* < 0.001) (Fig. 8 and Table 2). However, there were no significant differences in TV% between the INC and DCMNC groups (DCMNC: 47.1 ± 17.3 %, *p* = 0.210). In addition, the value was statistically similar between the DCM group and the controls.

The mean trabeculated myocardial volume in INC group (32.1 ± 13.8 mL) was 1.4 times lower than in NINC group (45.1 ± 15.3 mL, *p* = 0.001), 1.9 times lower than in DCMNC group (61.6 ± 25.5 mL, *p* < 0.001), 1.3 times lower than in DCM group (41.9 ± 18.4 mL, *p* = 0.01), and 1.3 times higher than in the controls (23.9 ± 6.9 mL, *p* = 0.007) (Table 2). In INC group, the trabeculated myocardial volume index (18.9 ± 7.1 mL/m^2^) was 1.4 times lower than in NINC group (26.3 ± 9.2 mL/m^2^, *p* = 0.001), 2.0 times lower than in DCMNC group (36.9 ± 15.6 mL/m^2^, *p* < 0.001), 1.3 times lower than in DCM group (25.0 ± 10.4 mL/m^2^, *p* = 0.008) and 1.4 times higher than in the control group (13.6 ± 3.7 mL/m^2^, *p* = 0.001).

We were able to measure trabeculated LV volume in all cases with an excellent interobserver reproducibility, and the intra-class correlation coefficient (ICC) for inter-rater reliability was 0.95 [95 % confidence interval (CI): 0.89–0.98]. There was a slight bias (1.8 ± 4.7), but no tendency. In addition, only one value out of 20 was outside the limits of agreement. There was also excellent intraobserver reproducibility. The ICC for intra-rater reliability was strong, with a value of 0.98 (95 % CI: 0.96–0.99).

The intraclass coefficients for intraobserver variability were 0.98 and 0.99 for LVESV and LVEDV, respectively, while the intraclass coefficients for interobserver variability were 0.95 and 0.88 for LVESV and LVEDV, respectively.

### Value of the percentage of trabeculated LV volume and semi-quantitative methods in LVNC diagnosis

**Fig. 9:**
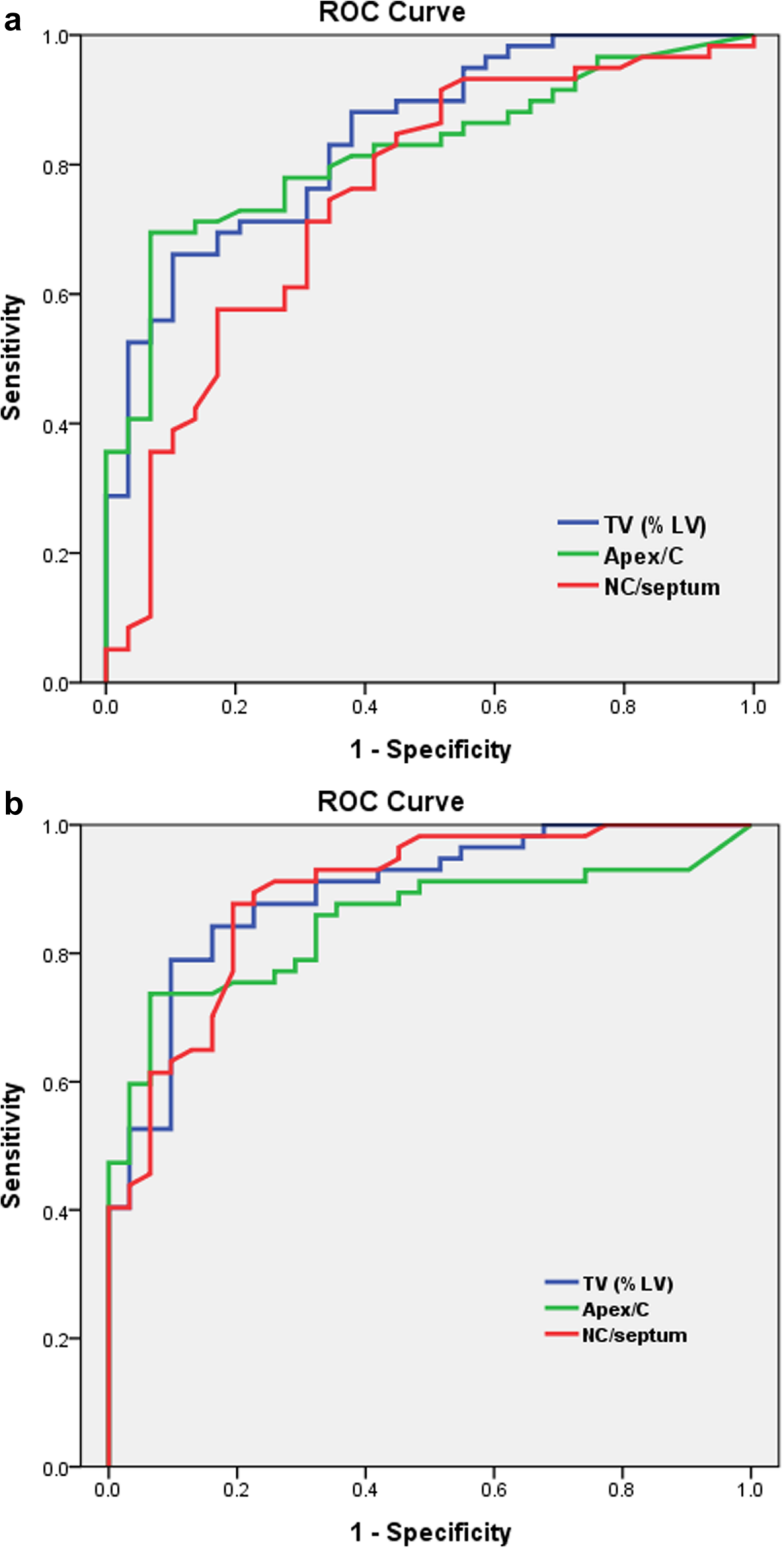
Receiver operating characteristic curves, using patient classifications according to Jenni’s method for CMR (**a**) and Petersen’s CMR criteria (**b**). The graphs describe the performance of the percentage of the trabeculated myocardial volume [TV (%LV)], the apex/C ratio, and the NC/septum ratio for LVNC diagnosis. Refer to Table 3 for details

The results of the ROC analysis for TV%, the apex/C ratio, and the NC/septum ratio for LVNC diagnosis using Jenni’s method for CMR classification or Petersen’s CMR classification as the standard diagnostic criteria were presented in Table 3 and Fig. 9. We also found that TV% was positively correlated with Jenni’s (*r* = 0.484 [95 % CI: 0.349–0.60], *p* < 0.001) or Petersen’s CMR criteria (*r* = 0.555 [95 % CI: 0.431–0.659]; *p* < 0.001) for all groups. In four patients with ventricular arrhythmia, their mean TV% was 43.9 % (range, 33.6 %–54.8 %) and their mean Petersen’s criteria measurement was 2.87 (range, 2.34–3.55).

**Table 3:** Performance of the percentage of the trabeculated myocardial volume, the apex/C ratio, and the NC/septum ratio for left ventricular non-compaction diagnosis from receiver operating characteristic curve analysis according to Jenni’s method for CMR and Petersen’s CMR criteria

Refined CMR criteria		Standard references	Area under curve [CI]	Specificity (%)[CI]	Sensitivity (%)[CI]
TV (%LV)	34.6 %	Jenni’s	0.843 [0.760-0.926]	89.7 [72.6 - 97.8]	66.1 [52.6 - 77.9]
	32.3 %	Petersen’s	0.894 [0.826-0.962]	90.3 [74.2 - 98.0]	79.0 [66.1 - 88.6]
Apex/C ratio	3.15	Jenni’s	0.820 [0.732-0.907]	93.1 [77.2 - 99.2]	69.5 [56.1 - 80.8]
	3.11	Petersen’s	0.849 [0.769-0.930]	93.6 [78.6 - 99.2]	73.7 [60.3 - 84.5]
NC/septum ratio	1.27	Jenni’s	0.750 [0.638-0.863]	82.8 [64.2 - 94.2]	57.6 [44.1 - 70.4]
	1.05	Petersen’s	0.892 [0.822-0.962]	80.7 [62.5 - 92.5]	87.7 [76.3 - 94.9]

*C* compacted, *apex* apical trabeculation thickness, *NC* non-compacted, *septum* septal wall thickness, *TV (%LV)* percentage of trabeculated myocardial volume over left ventricular myocardial volume

The mean time required for the trabecular volume measurement was about three minutes (range: two to five minutes).

## Discussion

The results of the present study can be summarized as follows. Trabeculated LV volume was significantly higher in INC patients as compared to healthy controls and DCM patients, although there was some overlap between the three groups. Patients with a TV% above 35 % could be considered as LVNC cases. Apex/C and NC/septum ratios could be supplemental diagnostic tools for LVNC, while LGE findings were rarely shown in the INC group. In DCMNC patients, trabeculated LV volume was positively correlated with LVEDV, LVESV, and the number of LGE segments, and it was negatively correlated with LVEF.

### Quantification of left ventricular trabeculation using CMR

The quantification of left ventricular trabeculation is an ideal method to diagnose INC in that it reflects the total volume of trabeculation and it does not depend on particular slices selected from the CMR images. The quantification of LV mass and volume with or without LV trabeculation has already been described and validated [19, 20]. Therefore, attempts to quantify trabeculation for the diagnosis of LVNC have already been performed by investigators [14, 21, 22]. The percentage of trabeculated LV mass over 20 % was suggested as a diagnostic criterion for INC by Jacquier et al. [14]. In addition, a maximal apical fractal dimension over 1.3, which reflects the complexity of the trabeculation, was proposed as a diagnostic criterion for INC by Captur et al. [21]. Recently, Grothoff et al. suggested that the percentage of non-compacted LV mass over 25 % should be the cutoff value for the diagnosis of LVNC, which differs from Jacquier’s cutoff value [22]. The results of our study were also inconsistent with these results.

In our study, TV% was significantly higher in the INC group compared to the healthy control and DCM groups. However, there was some overlap between the three groups. Interestingly, TV% in the DCM group showed a broad overlap with that of the INC group; these findings are in contrast to the results of Jacquier et al. [14].

### Supplementary diagnostic tools for LVNC

Petersen et al. excluded the measurement of the apex (segment 17), because the apical compacted myocardium is generally thinner than other segments, and its inclusion would have led to an artificially high NC/C ratio [13]. However, there are many studies that show hypertrabeculation is commonly involved in the apical segment [13–15]. In our study, the apical segment was involved in about 84 % of INC patients, while, in 6.2 % (9/145) of subjects, the apex/C ratio could not be used because of a lack of apical trabeculation.

In extremely non-compacted cases, compacted myocardium was difficult to measure for the calculation of various ratios, especially Petersen’s criteria. The thickness of compact myocardium in the area of LVNC was less than 2 mm in most patients (>66 %) except the control group. The value of the non-compacted myocardium to septal wall thickness might be useful in these cases.

### Myocardial fibrosis in LVNC

LGE enables noninvasive assessment of cardiomyopathy for the detection of myocardial fibrosis [23, 24]. In our study, only 4.3 % of INC patients presented with LGE. Our findings are consistent with other studies that also noted a lack of LGE in LVNC patients [22, 25]. However, there have also been studies that reported a high prevalence of LGE findings [26, 27]. According to Wan et al. [27], LGE was present in 40 % of 47 patients with LVNC. The reason for these discordances is unclear. Grothoff et al. [22] pointed out that the contradictory LGE findings might be caused partially by differences in imaging techniques rather than by differences in the distribution of age and LVEF. Burke et al. found from their pathological study that all 14 of the cases included showed marked endocardial fibrosis consistent with microscopic infarcts, and that areas of subendocardial replacement fibrosis were present in patients older than three weeks [28]. At present, further studies with a larger sample size are needed to investigate the pathophysiology and significance of LGE in INC.

### Dilated cardiomyopathy with LVNC

In the DCMNC group, we found that trabeculated LV myocardial volume was positively correlated with LVEDV and LVESV and negatively correlated with LVEF. In the DCM group, however, there was no correlation between trabeculated LV volume and LVESV or LVEF. In our study, there was also a positive correlation between trabeculated LV myocardial volume and the number of LGE segments in the DCMNC group. It has already been reported that myocardial fibrosis predicts a poor prognosis in DCM patients [29, 30]. Masci et al. also found that the absence of LGE at baseline is a strong independent predictor of LV reverse remodeling in idiopathic DCM patients [29]. Therefore, we conclude that it is important to detect LVNC in DCM patients, because DCMNC patients might have decreased LV systolic function and an increased prevalence of LGE findings.

### Limitations

This study has some limitations that should be acknowledged. First, it was a retrospective study. Second, because there was no standard reference for LVNC, Jenni’s method and Petersen’s CMR criteria were used to define our LVNC cases. Third, a selection bias may have been introduced, as the study population included patients presenting to a tertiary referral center. Fourth, although we excluded the papillary muscles from the measurement, we admit that poorly formed papillary muscle could be counted as trabeculation. The patients’ genetic factors were not considered in the LVNC diagnoses. A genetic factor could help to provide more accurate diagnoses for LVNC and would provide a more powerful means of validating our method. We did not use trabecula-dedicated software to measure the trabecular myocardial volume excluding the blood cavity. However, the manual measurement of trabecular volumes using PACS or workstations provided by MR equipment vendors was neither time-consuming nor difficult in our study. Future studies are expected to use some trabecula-specific software.

## Conclusion

In this study, we used cardiac magnetic resonance imaging to establish refined diagnostic criteria for LVNC. As a quantitative approach, we have shown that a TV% > 35 % of the LV myocardial volume is diagnostic for LVNC with high specificity. In addition, as a semi-quantitative approach, we propose a reproducible method of using apex/C and NC/septum ratios for supplemental diagnostic criteria for LVNC.

## Abbreviations

C: compacted
CAD: coronary artery disease
CHF: congestive heart failure
DCM: dilated cardiomyopathy
DCMNC: dilated cardiomyopathy with non-compaction
INC: isolated left ventricular non-compaction
LGE: late gadolinium enhancement
LV: left ventricular
LVEDV: left ventricle end-diastolic volume
LVEDVi: left ventricle end-diastolic volume/body surface area
LVEF: left ventricle ejection fraction
LVESV: left ventricle end-systolic volume
LVESVi: left ventricle end-systolic volume/body surface area
TV%: the percentage of trabeculated LV volume relative to total LV myocardial volume
